# Household Chaos and Child Behavior Problems Predict Maternal Well-being

**DOI:** 10.1007/s11126-021-09947-2

**Published:** 2021-09-01

**Authors:** Sumeyra Yalcintas, Alison Pike, Bonamy R. Oliver

**Affiliations:** 1 School of Psychology, University of Sussex, Brighton, BN1 9RH UK; 2 Dept Psychology and Human Development, UCL Institute of Education, University College London, London, UK

**Keywords:** Maternal well-being, Household organization, Household chaos, Child behavior problems

## Abstract

The aim of the study was to investigate predictors of maternal well-being in mothers of twins. As well as being important in its own right, maternal well-being is a crucial predictor of parenting (Belsky in Child Dev. 55(1):83, 1984). Based on previous research (Pike et al. in Int J Beh Dev. 30(1):55–66, 2006) we expected that household chaos (Confusion, Hubbub, and Order) and child behavior problems would predict maternal depression, stress and anxiety. The data for the study was taken from the Twins, Family and Behavior Study (TFaB) -- a longitudinal UK study of twins born in 2009 and 2010. One hundred and fifty-eight mothers of twins (*M*_child age_= 6.01 years, *SD*_*age*_* =* 0.50) reported on household chaos, child disruptive behaviors and their own well-being. Higher levels of household chaos were linked to maternal depressive, anxiety and stress related symptoms. More child behavior problems were related to more depressive and stress symptoms but not anxiety. The findings show promise for future research investigating different types of maternal well-being and suggested practical implications, such as intervening on concrete aspects of household chaos to improve maternal well-being.

Maternal well-being (e.g., depression, stress and anxiety) is critical both for mother and child. The present study hypothesized that household disorganization would predict maternal well-being based on previous research [2] and further tested child behavior problems as another predictor. The aim of the study was to examine relationships between household chaos, child behavior problems and maternal well-being, and to uncover patterns of differentiation for well-being. The sample consisted of families with twins,such families require more organization and effort in parenting. Specifically, we investigated whether household chaos and child behavior problems predicted maternal depression, stress and anxiety in similar or distinctive ways.

## Maternal Well-being

Studying maternal well-being is important, not only for mothers, but also because it is predictive of better parenting. For example, better maternal mental health predicts better quality parenting [1] and secure child attachments [3]. Conversely, depressed mothers are more hostile [4], demonstrate fewer sensitive behaviors [5] toward their children, and have more negative interactions with their infants [6]. Therefore, studying predictors of maternal well-being is important for understanding the mother-child relationship. Importantly, existing studies largely investigate one aspect of maternal well-being (e.g., depression) or several aspects as a composite (e.g., depression, stress and anxiety in combination). However, there are theoretical reasons to consider differentiating well-being (studying depression, anxiety and stress individually). The Tripartite Model of Anxiety and Depression [7] has been used to explain comorbidity of depression and anxiety, dividing them into physiological arousal and positive and negative affect. Studies investigating mothers during the prenatal and postnatal periods have demonstrated distinct findings for maternal anxiety and depression. For example, children’s internalizing problems are influenced by maternal depression but not by anxiety [7]. Yet the research on differentiation of maternal well-being is limited. To address this gap, we defined maternal well-being as the level of depression, stress and anxiety symptoms reported by mothers, aiming to uncover patterns of differentiation for well-being.

## Chaos, Child Behavior Problems, and Maternal Well-Being

Homes low in regularity, and high in noise and crowding, have been defined as high in household chaos [8]. Higher levels of household chaos have been related to maternal depression and stress [2], as well as with unsupportive behaviors of mothers toward their children [9]. The role of child behavior problems has also been studied in relation to maternal well-being. For example, children of depressed mothers have more behavior problems than children with non-depressed mothers [10]. Although such associations are commonly conceptualized as parent to child effects, there is also evidence of bidirectional effects,child characteristics can also influence parents. Research on children’s temperament, for example, demonstrates that mothers of children with difficult temperaments are at risk of depression and stress [11–13].

## The Present Study

The sample for the current study was families with twins, families that typically experience additional stress. Given the links between household chaos, maternal well-being, parenting and child behavior problems, we hypothesized a model whereby household chaos and child behavior problems predicted maternal well-being. Previous research indicates that household chaos is associated with conduct problems, impulsivity and delinquency [14, 15], externalizing behaviors and aggression, [16] and diverse child adjustment problems [17, 18]. Due to the probable correlation among our predictors, we investigated independent predictions from household chaos and child behavior problems to maternal well-being, as well as examining their prediction as a whole. Specifically, higher levels of household chaos and child behavior problems were hypothesized to predict higher levels of depression, anxiety and stress. Additionally, we aimed to uncover possible differentiation of these aspects of maternal well-being.

## Method

### Participants and Procedure

Data was drawn from the Twins, Family and Behavior Study (TFaB) -- a longitudinal UK study of twins born in 2009 and 2010, that investigates family relationships and children’s behaviors. The data for this study was cross-sectional. In total, 158 mothers (*M*_age_= 38.14 years; *SDage =* 4.32 years) of twins reported on levels of household chaos, child behavior problems and their own symptoms of depression, anxiety and stress, (*M*_child age_= 6.01 years, *SDage =* 0.50 years). Fifty-four of the twins were monozygotic and 101 were dizygotic, with 3 pairs of unclassified zygosity. One-hundred-and-forty-three (91%) mothers were married or cohabiting with a partner. Mothers were highly educated, 71.3 % of the sample reported having an undergraduate or higher degree, and only 0.6% of mothers reported having no qualification. Ethnicity information was not collected in this sample. Household income ranged between less than £5,000 and more than £100,000, with a median response of £40,000- £49,000. Mothers completed postal questionnaires for all study variables.

### Measures

#### Household Chaos

 The short version of the Confusion, Hubbub, and Order Scale (CHAOS; [10]) was used to assess household chaos, and consisted of 6 items. Mothers rated the items on a 5-point scale (1 = *definitely untrue*, 5 = *definitely true*). Example items are “The children have a regular bedtime routine (e.g., same bed each night, a bath before bed, reading a story) ” (reverse-scored) and “It is a real zoo in our home”. The Cronbach’s α was .67.

#### Child Behavior Problems

 The Eyberg Child Behavior Inventory (ECBI; [19]) was given to mothers to assess child behavior problems. The response format was a 7-point Likert type scale (1= *never*, 7= *always*) and the questionnaire has 36 items. Example items are “Destroys toys and other objects” and “Acts defiant when told to do something”. Items were summed, with higher scores indicating more disruptive behaviors. The correlation between maternal reports of their twins’ behavior problems was *r* = .81. Due to this substantial twin similarity, we created a single variable for each family. The Cronbach α for this measure was .93.

#### Maternal Well-Being 

The Depression Anxiety Stress Scales (DASS-21; [20]) was used to measure maternal well-being. The questionnaire has 3 sub-scales, each of seven items (note that in the current study one item measuring depression was inadvertently missing). Mothers were asked to rate how much each statement applied to them over the past week on a 4-point scale (0 = *did not apply to me at all*, 3= *applied to me very much or most of the time*). A sample item for depression is “I felt that I had nothing to look forward to,” for anxiety “I felt I was close to panic,” and for stress “I found it difficult to relax.” Scores for each subscale were summed and multiplied by 2. Cronbach’s α values were good for depression (*α* = .82) and stress (*α* = .77) subscales, and reasonable for anxiety (*α* =.62).

## Results

### Preliminary Analysis

Table 1 contains correlations and descriptive statistics among all study variables. As expected, higher levels of chaos were related to more depression, stress and anxiety symptoms, and also to more child behavior problems (*r* = .39, *p* < .001). More child behavior problems were related to more maternal depression (*r* = .31, *p* < .001), and stress (*r* = .31, *p* < .001) but not more anxiety. Socioeconomic status, maternal age, educational level and zygosity of twins were not related with maternal well-being, so we did not control for these variables in the main analysis.

**Table 1:** Descriptive Statistics & Bivariate Relations

Variable	*1*	*2*	*3*	*4*	*5*
1. CHAOS		.32**	.23*	.29**	.39**
2. Depression			.61**	.54**	.31**
3. Anxiety				.44**	.15
4. Stress					.31**
5.Child Behavior Problems					
Mean (SD)	2.39 (.63)	3.52 (4.39)	2.82 (3.61)	9.92 (5.63)	3.10 (.62)
Range	1.17-4.00	0-30	0-20	0-32	1.66-5.14

* *p* < .05; ** *p* < .001

### Multiple Regression Analysis

To understand independent contributions of household chaos and child behavior problems to maternal well-being, three multiple regression analyses were conducted (see Table 2). For depression, the entire model was significant *F* (2, 154) = 12.41, *p* < .001, explaining 13% of the variation. Both chaos (*B* = .23, *t* = 2.86, *p* < .05) and child behavior problems (*B* = .21, *t* = 2.62, *p* < .05) significantly predicted depression. However, for anxiety, the results were slightly different. The overall model was significant *F* (2, 154) = 4.49, *p* < .05, explaining 5% of the variation. The only significant predictor was chaos (*B* = 19, *t* = 2.32, *p* < .05). Finally, for stress the model was very similar to depression, *F* (2, 154) = 11.33, *p* < .001, explaining 12% of the variation, with both the predictors chaos (*B* = .19, *t* = 2.35, *p* < .05) and child behavior problems (*B* = .23, *t* = 2.88, *p* < .05) providing significant prediction.

**Table 2:** Multiple Regression Models for 3 Well-being Variables

	*t*	*p*	*B*	*F*	*df*	*p*	*Adj R*^*2*^
*Depression*							
Overall Model				12.41	154	.000	.13
Chaos	2.86	.005	.23				
CBP	2.62	.010	.21				
*Anxiety*							
Overall Model				4.49	154	.013	.04
Chaos	2.32	.021	.20				
CBP	.84	.405	.07				
*Stress*							
Overall Model				11.33	154	.000	.12
Chaos	2.35	.020	.19				
CBP	2.88	.004	.24				

CBP is Child Behavior Problems. Standardized B values are reported

*N* = 156

## Discussion

We used a UK- based sample of families with young twins, to test household chaos and child behavior problems as predictors maternal well-being. There was an advantage of using a twin sample in that families with twins require more organization and effort in parenting, and previous studies have used twin samples to understand household chaos and child behavior problems [21–24]. Mothers perceiving more household chaos tended to report more depressive, stress and anxiety related symptoms. More child behavior problems also predicted more depressive and stress related symptoms, but it was not predictive of maternal anxiety**.** The results supported previous research indicating household chaos correlates with maternal well-being [2]. Notably, the results showed differentiation of maternal well-being in relation to child behavior problems. This highlights the importance of studying these aspects of maternal well-being separately.

We also demonstrated the independent prediction of chaos and child behavior problems with maternal well-being outcomes, in the context of existing literature that shows a link between child behavior problems and chaos [16–18]. The results indicate when chaos and child behavior problems put in the model together more chaos was a significant predictor of all well-being variables but for child behavior problems it was depression and stress. Results demonstrated that if mothers perceive more chaos and disorganization in the home, they may be at risk of having more depression, stress symptoms and anxiety. If they report more child behavior problems, they may also be at risk of having more depression and stress but not anxiety. These findings suggest that interventions focused on reducing the levels of chaos in the home, (e.g. implementing a regular bedtime routine, inducing calm and having an organized schedule) and/or reducing child behavior problems may also result in improved maternal well-being. The results can also imply that child behavior problems are risk factors for poor maternal well-being, highlighting the bi-directional influence mothers and children have on one another.

### Limitations

Despite the strengths of our novel study, we acknowledge some limitations. The current sample was mothers of twins and requires replication across different family types. Additionally, the sample was highly educated, 71.3 % of the mothers reported having an undergraduate or higher degree, so the results cannot be generalized to all populations. Future studies should investigate whether the same pattern of results are apparent not only for parents of non-twin children, but also more representative samples in terms of education levels. Another point that worth mentioning is that the anxiety subscale showed poor internal reliability, which may have attenuated associations. Most important, a longitudinal design with all measures repeated over time would enable cross-lagged models to identify the temporal ordering of influence.

## Conclusion

The current study showed that chaos predicted maternal depression, anxiety and stress, whereas child behavior problems were predictive of maternal depression and stress – but not anxiety. In order to have better outcomes for maternal well-being as well as for parenting, household chaos and child behavior problems should be targeted in interventions. The results suggest that child behavior problems and chaos are risk factors for impaired parental functioning.
